# Applying the equivalent uniform dose formulation based on the linear‐quadratic model to inhomogeneous tumor dose distributions: Caution for analyzing and reporting

**DOI:** 10.1120/jacmp.v1i4.2634

**Published:** 2000-09-01

**Authors:** John. E. McGary, Walter Grant III, S. Y. Woo

**Affiliations:** ^1^ Department of Radiology Baylor College of Medicine Houston Texas 77030

**Keywords:** equivalent uniform dose, linear quadratic, intensity modulated radiotherapy

## Abstract

We apply the concept of equivalent uniform dose (EUD) to our data set of model distributions and intensity modulated radiotherapy (IMRT) treatment plans as a method for analyzing large dose inhomogeneities within the tumor volume. For large dose nonuniformities, we find that the linear‐quadratic based EUD model is sensitive to the linear‐quadratic model parameters, α and β, making it necessary to consider EUD as a function of these parameters. This complicates the analysis for inhomogeneous dose distributions. EUD provides a biological estimate that requires interpretation and cannot be used as a single parameter for judging an inhomogeneous plan. We present heuristic examples to demonstrate the dose volume effect associated with EUD and the correlation to statistical parameters used for describing dose distributions. From these examples and patient plans, we discuss the risk of incorrectly applying EUD to IMRT patient plans.

PACS number(s): 87.53.Tf

## INTRODUCTION

Currently, there is a significant number of radiation therapy departments that are actively implementing and using intensity modulated radiotherapy (IMRT). The goal of these systems is to shape the dose distributions to the tumor volume and reduce the dose to healthy tissue and sensitive structures to a greater extent than possible with unmodulated beams. The resultant dose distribution and extent of tumor dose nonuniformity depends on the dose optimization algorithm, treatment delivery device, planning method, and the geometric relation between the critical structures and target. Optimization algorithms differ in the degree of tumor dose nonuniformity they induce, which is partially determined by the scoring scheme or criteria used to select the radiation beams.^1^ Some optimization systems, like the PeacockPlan (NOMOS Corporation, Sewickly, PA), minimize the sum of the squared tumor dose residuals which allows for zero dose to a small part of the target volume and can produce a large dose nonuniformity.^2^ For conditions where a critical structure lies adjacent to the tumor, a substantial dose inhomogeneity may exist, depending on the penalty associated with the structure. To exacerbate this condition, treatment delivery devices like the Peacock intensity modulated multileaf collimator (MIMiC) induce dose inhomogeneity due to the field matching problem within the tumor.^3^ Field junctioning within the tumor may produce regions of reduced dose with respect to the surrounding target volume, and in these instances, the matchline is usually associated with the minimum dose to the target. This presents problems for planning evaluation since the bulk of experience is with fairly uniform dose distributions, where typically, plans contain large fields with small dose deficits that occur at or near the periphery of the target volume.

Better methods to report and analyze IMRT treatment plans are needed due to the uncertainties in clinical outcome as a result of tumor dose. With three‐dimensional (3D) treatment planning systems, there is a vast amount of data detailing the patient anatomy and dose distribution within the irradiated volume. Summarizing 3D dose distributions is essential for evaluating plans and performing outcome analysis: reports need to be concise with sufficient detail to permit relevant analysis and comparisons. However, without knowing the relevant parameters for analysis, summarization reduces the dimensionality and detail of information which may prevent crucial correlations. Cumulative and differential dose‐volume histograms (CDVH/DVH) provide a convenient method for reducing a large amount of 3D data while retaining dose‐volume detail but are deficient in location correlations, and furthermore, the dose‐volume information is not concisely summarized for reporting or plan evaluation.^4^ ^,^ ^5^ At the other extreme of dose summarization, dose statistics, (e.g., minimum, maximum, and mean dose) are single numbers that characterize certain aspects of the 3D dose distribution and greatly reduce the information for analysis; more parameters are required to increase the dimensionality and allow for adequate analysis. Dose statistics are meaningful descriptions for conditions supported by strong clinical experience and easily used for plan evaluation, whereas in situations where there is little experience, dose statistics may limit the possibility for adequate analysis. In contrast to dose statistics and dose‐volume data, biological models attempt to translate dose‐volume information into estimates of biological impact. Unfortunately, these models are not accepted as accurate outcome predictors, and at this time, models for tumor control probability (TCP) may only be useful for scoring plans on a specific patient.^6^

Although patients are treated with large dose inhomogeneities, a general method and the necessary parameters for analysis has not been agreed upon. In the past, there have been some suggestions. For example, Brahme proposed that the effective dose delivered to the target can be approximated by the mean target dose for small dose inhomogeneities and that the minimum target dose should be used for large dose nonuniformities.^7^ The Nordic Association of Clinical Physics (NACP) recommended that the arithmetic mean value of the target and the standard deviation of the dose distribution should be used for dose prescription and reporting.^8^ Recently Niemerko proposed a quantitative method for reporting and analyzing inhomogeneous dose distributions. Niemerko used the equivalent uniform dose (EUD) concept, based on the linear‐quadratic (LQ) model, and stated that EUD should be a better single indicator of radiotherapy outcome than other measures commonly used.^9^

To understand our dose distributions generated from the Peacock treatment planning system, we used the LQ‐based EUD model to analyze our patient plans and discovered conclusions that contradict those of Niemerko. Niemerko's study concluded that EUD was insensitive to the biological model parameters and that only a single EUD is needed to describe a non‐uniform dose distribution. In contrast, we found that EUD is sensitive to the biological parameters and interpreting these dose distributions requires more analysis than calculating a single EUD.

In this paper, we demonstrate, through simple examples, that EUD is not, in general, independent of the linear‐quadratic model parameters. We further show that this variability is a function of the model parameters and that treatment plans may be analyzed incorrectly without considering this problem. We discuss the caution that must be exercised when using EUD.

## METHODS

For tumors, the concept of equivalent uniform dose (EUD) is based on the assumption that different dose distributions are equivalent if the corresponding expected number of surviving clonogens is equal. For any dose distribution, EUD is the homogeneous dose to the target that produces the same number of clonogens as the nonuniform dose distribution delivered to the identical target. It is assumed that a tumor is composed of a large number of independent clonogens and that the random killing of cells can be described by Poisson statistics. In addition, it is assumed that the surviving fraction, SF(*d*), of cells irradiated to a homogeneous dose is modeled by the linear‐quadratic model

(1) $$\text{SF}(d)=e^{-\alpha d-\beta d^{2}}$$

where α and β refer to the two components of cell killing, irreparable and reparable damage.^10^ For fractionation effects, the surviving fraction can be described as

(2) $$\text{SF}(n,D)=e^{-\left(\alpha D+\beta D^{2}/n\right)},$$

where *D* is the total dose delivered over *n* fractions.^11^ For an inhomogeneous dose distribution, $\left\{D_{j}\right\}$, the surviving fraction of tumor cells is found to be equal to

(3) $$SF(n,\{D_{j}\})=\sum_{j}v_{j}\exp\left(-\alpha D_{j}-\frac{\beta D_{j}^{2}}{n}\right),$$

where $D_{j}$ is the total dose delivered to the partial volume $v_{j}$ and *n* is the number of dose fractions. By equating the surviving fractions given in Eqs. (2) and (3), the equivalent uniform dose, EUD, is

(4) $$\text{EUD}=\frac{-n\alpha }{2\beta }\pm \frac{1}{2}{\left\{{\left(\frac{n\alpha }{\beta }\right)}^{2}-\frac{4n}{\beta }\times \ln\left[\sum_{j}v_{j}\exp\left(-\alpha D_{j}-\frac{\beta D_{j}^{2}}{n}\right)\right]\right\}}^{5}.$$

Equation (4) is essentially the same equation as presented by Niemerko where EUD is defined in terms of SF(2 Gy); however, we chose to use the nomenclature used in Refs. 10 and 11.

## RESULTS

**Figure 1:**
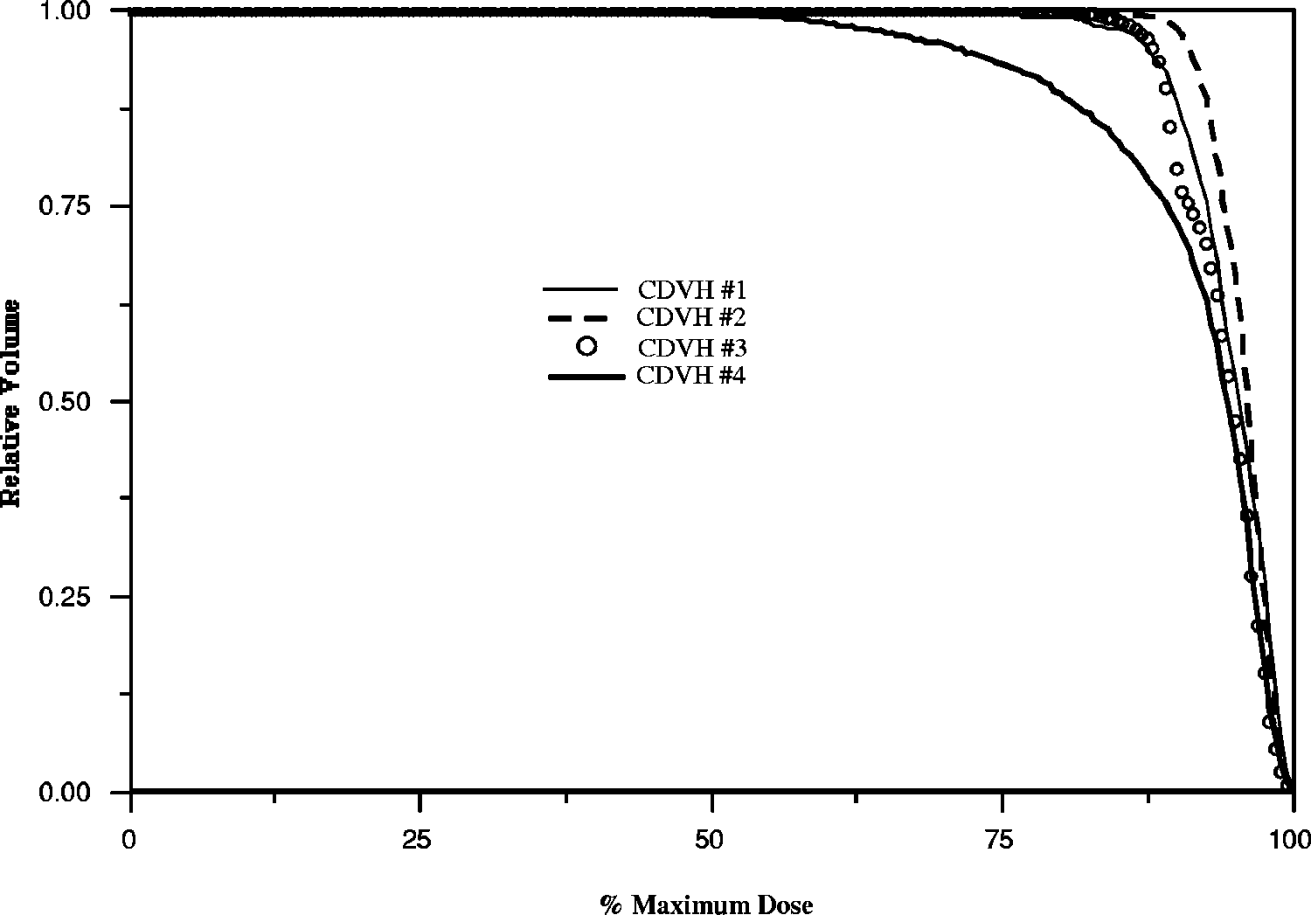
Cumulative dose volume histogram (CDVH) examples of clinically delivered Peacock plans. The abscissa is defined as percentage of maximum dose to the tumor. The four dose distributions (CDVH#) are summarized by the averaged equivalent uniform dose (EUD), mean dose (Gy), standard deviation (SD), the prescription dose at per cent of maximum dose, the dose per fraction (Gy/fx), and the minimum dose (shown in Table I).

Figure 1 illustrates the variety of clinically delivered Peacock patient plans that need to be considered for dose reporting and analysis. These figures represent the typical shape of the treatment plan CDVHs along with the variation in fractionation, prescription, and tumor dose nonuniformity. Within these examples shown in Fig. 1, the prescription dose ranges from 20 to 60 Gy and the dose per fraction extends from 2 to 4 Gy. Tumor dose nonuniformity for each plan is qualitatively described by the CDVHs, and the 3D dose distributions are further summarized by the standard deviation (SD), minimum, mean, and the equivalent uniform dose (EUD). The minimum dose is defined as the smallest dose and the maximum dose is the largest dose delivered to a voxel within the target volume. The EUD values shown in Fig. 1 are calculated from Eq. (4) for each CDVH and are averaged over a range of α from 0.1 to 0.5, with a fixed ratio of α/β=10.

**Table I:** Summary for Fig. 1.

CDVH #	EUD (Gy)	Mean (Gy)	SD (Gy)	Prescription	Gy/fx	Min (Gy)
1	21.4	21.5	0.86	20 Gy@88%	4	17
2	31.8	31.8	0.83	30 Gy@90%	3	27
3	53.2	53.4	2	50 Gy@88%	2	43
4	48.3	60.6	5.9	60 Gy@90%	2	28

Each plan has an inhomogeneous dose distribution but to varying degrees. In comparison with the goals set by ICRU 50, the four dose distributions presented in Fig. 1 exhibit large dose nonuniformities.^12^ The relative difference between the minimum and maximum dose to the prescription value exceeds 12% and the minimum dose is much less than 95% of the prescription — dose gradients within these tumor volumes are greater than about 25%. A better descriptor for the degree of dose inhomogeneity may be represented by the standard deviation. Even though the plans exhibit large dose inhomogeneities with respect to ICRU 50, the relative standard deviation as compared with the prescription dose is relatively small, less than 1%, for two plans. In the case of CDVH#4, the relative standard deviation is much larger, ~10%. For the purpose of this discussion, plans with standard deviations greater than 2 Gy for prescription doses larger than 60 Gy will be referred to as plans with large dose inhomogeneities.

**Figure 2:**
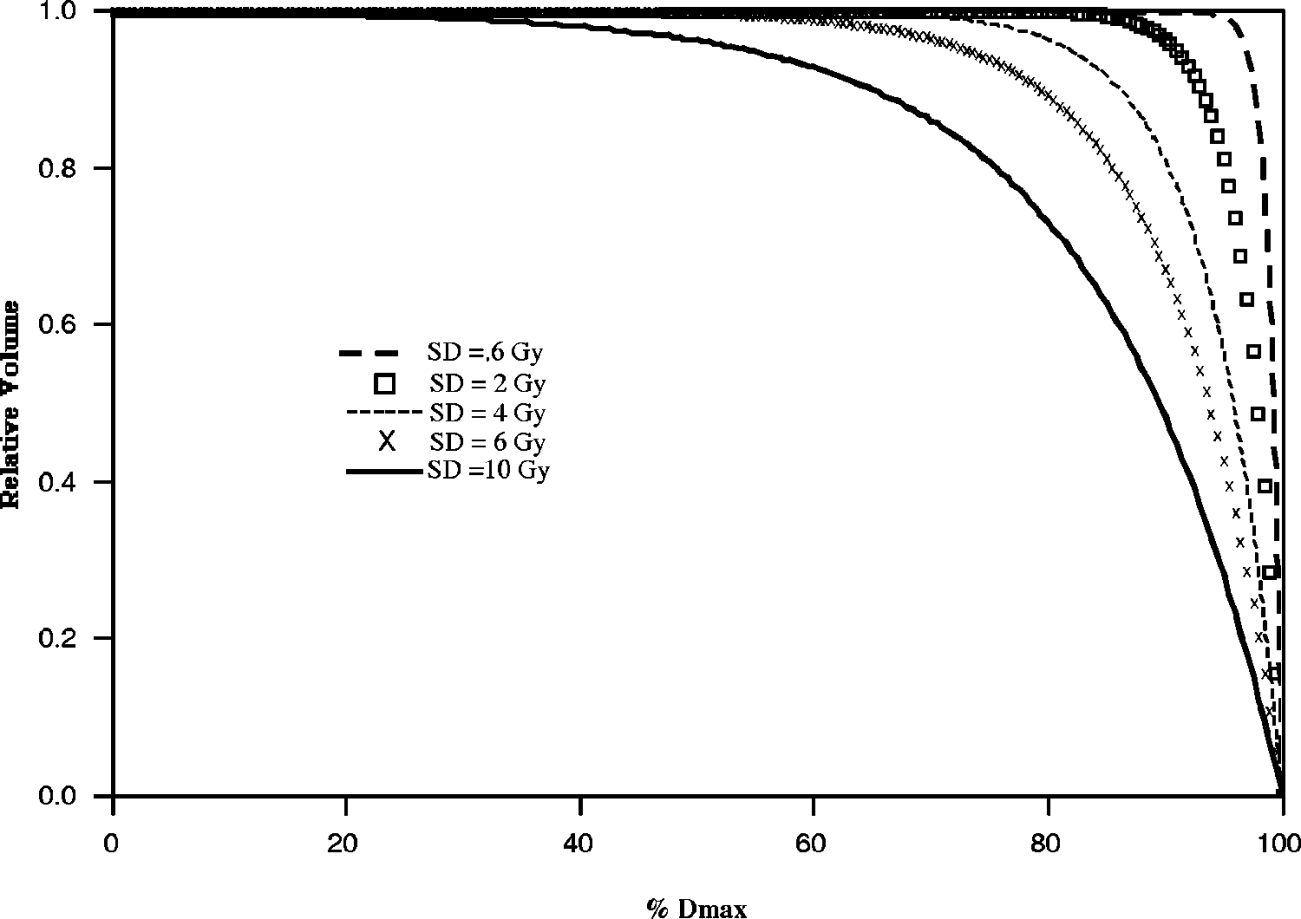
Generated CDVHs to model Peacock patient plans and characterized by the standard deviation (SD). The maximum dose is equal to 66.7 Gy.

**Figure 3:**
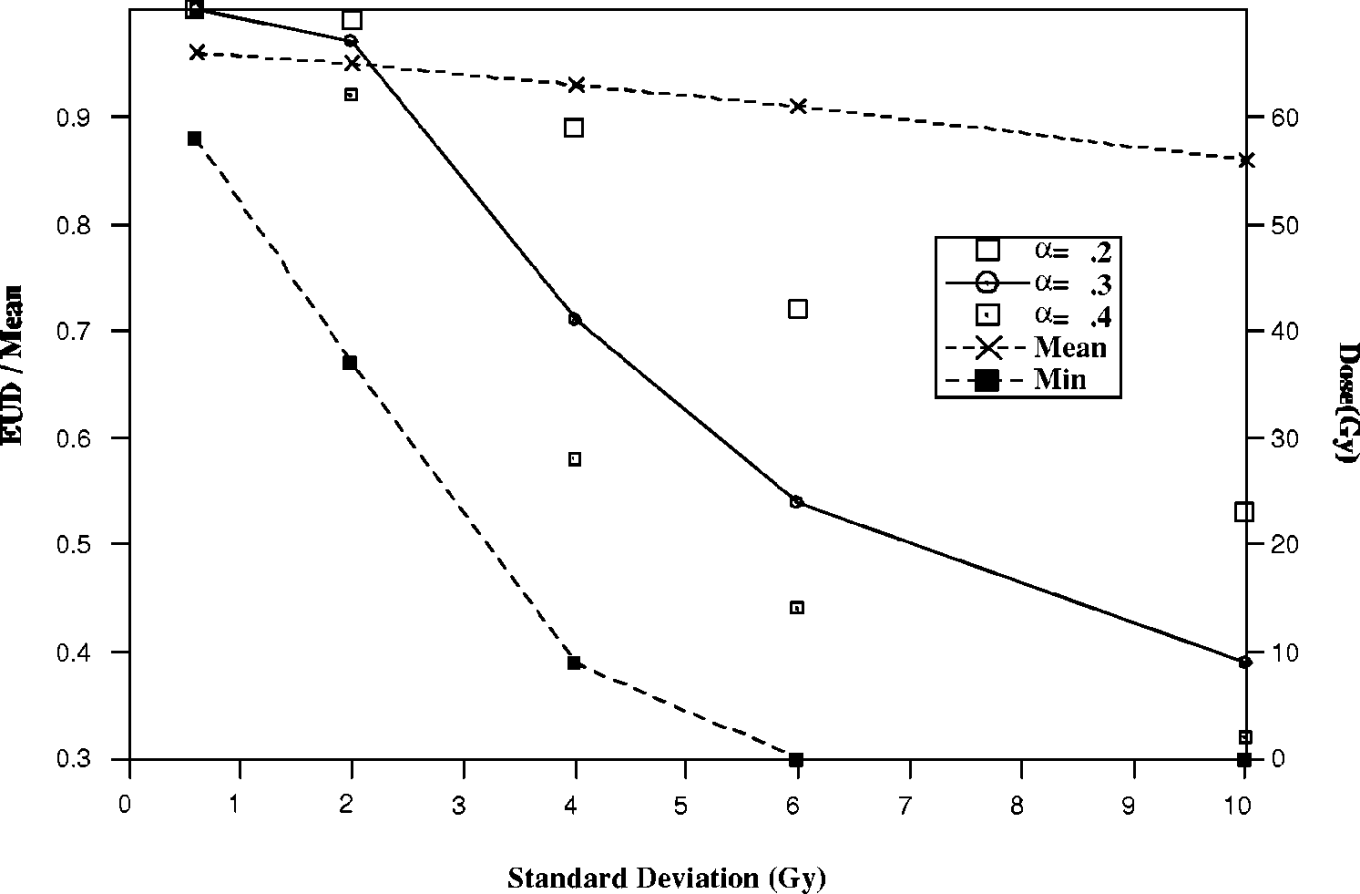
Results from example CDVHs indicating variability in equivalent uniform dose (EUD) with respect to the linear‐quadratic (LQ) parameters, α and β. The ratio of EUD to mean dose is plotted as a function of standard deviation for α=0.2, 0.3, and 0.4 with α/β=10 and 2 Gy per fraction. The mean and minimum dose in Gy are plotted as a function of standard deviation.

**Figure 4:**
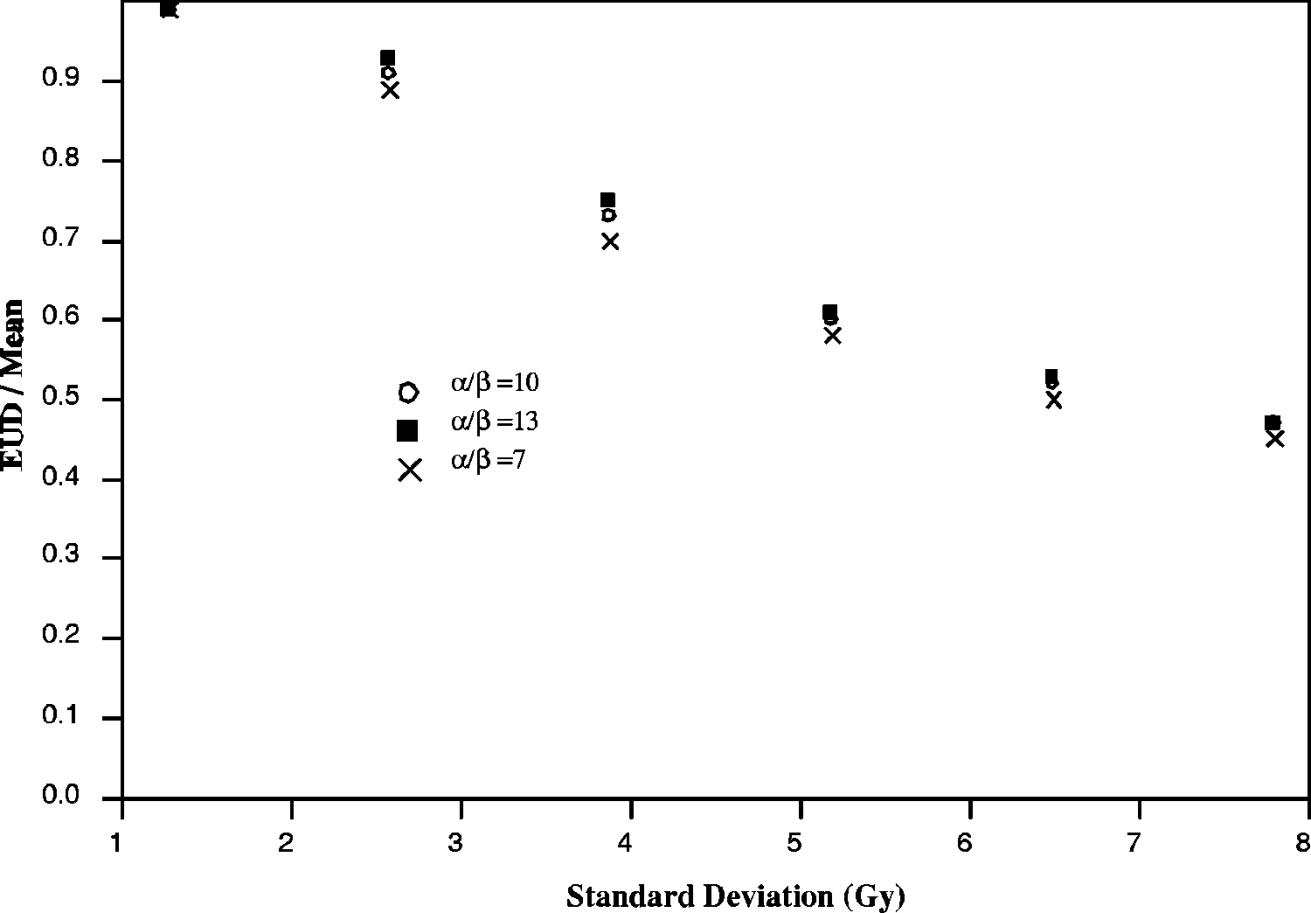
Variations in EUD with respect to ratios of α/β=7, 10, and 13 for α=0.3.

**Figure 5:**
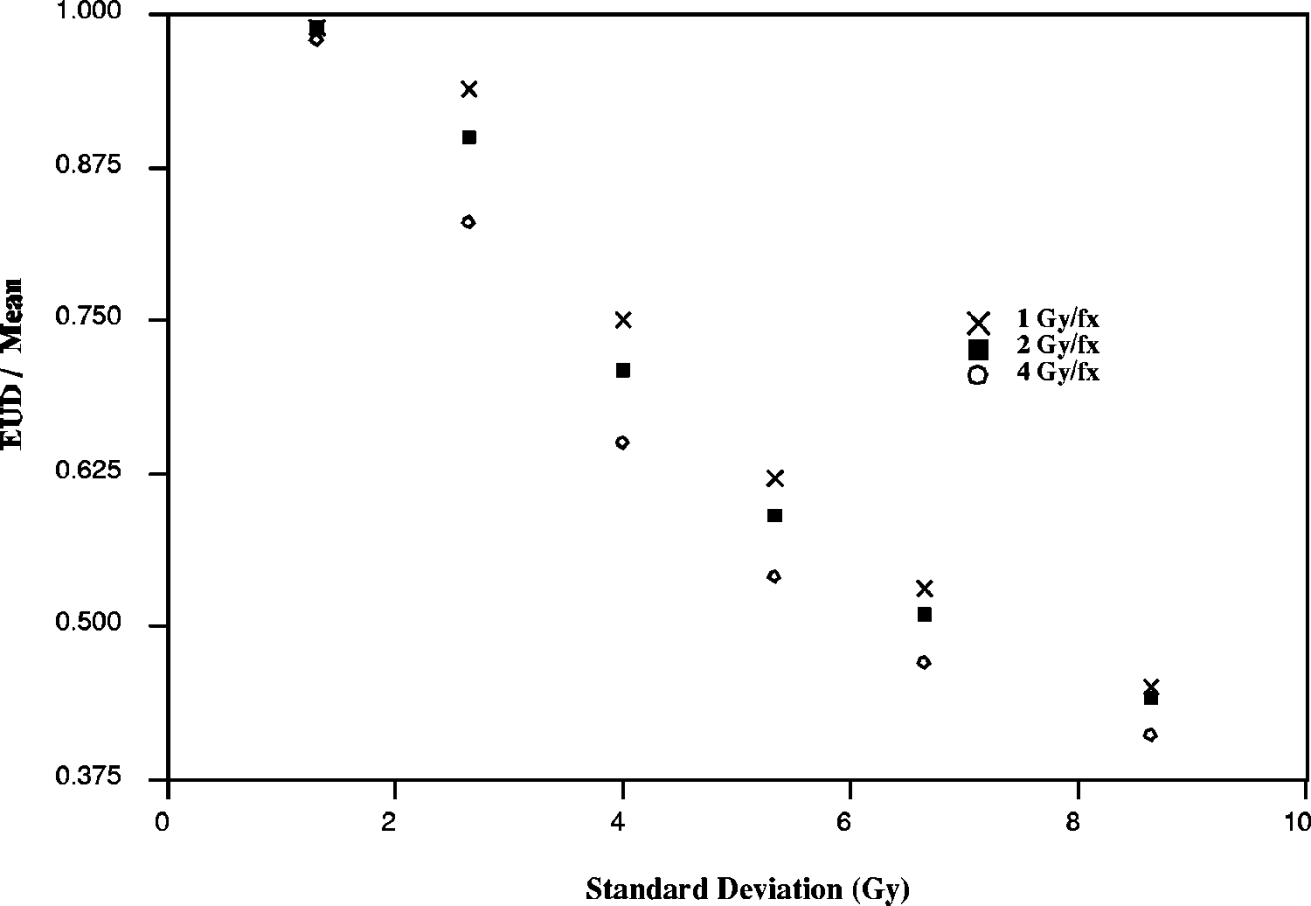
Variation in EUD with respect to fractionation dose equal to 1, 2, and 4 Gy per fraction. EUD is calculated with α/β=10 and α=0.3.

To investigate the relation of EUD with respect to dose inhomogeneity, a series of CDVHs were generated to model typical Peacock (Peacock Plan or CORVUS) patient plans (Fig. 2). Each cumulative dose volume histogram is characterized by a standard deviation (SD), to represent the degree of dose inhomogeneity, and plotted as a function of percent maximum dose, which is chosen to be 66.7 Gy. The range of standard deviation considered here is from 0.6 to 10 Gy. For each CDVH, the EUD is calculated over a range of linear‐quadratic parameters, α and β, and the results are shown in Figs. 3–5. Figure 3 demonstrates that EUD is a function of the model parameters. The variation in EUD is shown for α=0.2, 0.3, and 0.4. In addition, the arithmetic mean and minimum dose for the different dose distributions are plotted as functions of standard deviation with the ordinate scale on the right side of the graph. For small standard deviations less than 2 Gy, the EUD of the dose distributions are approximately equal to the mean dose. For increasing dose inhomogeneity, the ratio of EUD to mean dose decreases nonlinearly, and the variation in EUD with respect to α increases significantly. For standard deviations greater than 4 Gy, EUD varies by greater than 40% between α=0.2 and 0.4. As a side note, the trend in the ratio of EUD to mean dose seems to mimic the minimum dose behavior which suggests that the minimum dose is linearly related to EUD, for a particular α. Although EUD is sensitive to α, it is much less dependent on the ratio α/β. EUD is approximately constant over an applicable range of α/β for a given value of α. Figure 4 demonstrates that for a fixed value of α, α=0.3, the variation in EUD with respect to α/β, between 7 and 13, is small over a large range of standard deviation and minimum dose.

Fractionation effects are also considered as shown in Fig. 5. For each of the cumulative dose volume histograms, the equivalent uniform dose is calculated at 1, 2, and 4 Gy per fraction, for a total dose of 60 Gy. The result shows that EUD depends on the dose per fraction for standard deviation values greater than 2 Gy where EUD variations are on the order of 10%.

The model CDVHs are not intended to precisely duplicate the delivered Peacock patient plans; instead, they are used as a tool to gain some insight into the interrelationships of the statistical and biological parameters for large dose nonuniformities. These CDVHs loosely model our sample of Peacock patient plans in shape but the patient plans exhibit a larger minimum dose value for a reference standard deviation. At a relatively small standard deviation, 2 Gy, the model CDVH appears to qualitatively describe CDVH #3 in Fig. 1. Inspecting the minimum to maximum dose ratio for both histograms shows that the model histogram has a lower dose ratio of ~55% compared to the Peacock plan which is ~75%. For standard deviations greater than 6 Gy, the minimum dose of the model is 0 Gy indicating that a portion of tumor volume is unirradiated which is not the case for the clinical plans.

## DISCUSSION

In general, EUD is not independent of the linear‐quadratic model parameters as was concluded by Niemerko. The model CDVHs demonstrate that there are dose distributions where EUD is a function of the linear‐quadratic model parameters and a single EUD value would misrepresent the dose distribution. In terms of the previous examples, the EUD variation is significant for standard deviations larger than 2 Gy. The incorrect conclusion drawn by Niemerko is partly due to the limited data set used for his study. The tumor dose distributions delivered using the Peacock system considered in this paper are different than the data set composed of clinical and modeled distributions examined by Niemerko. Although both data sets exhibit large tumor dose inhomogeneities, the relative amount of volume under and overdosed is different between the two data sets. Qualitatively, the CDVHs analyzed by Niemerko are sigmoidal with hot and cold volume elements distributed symmetrically about the prescription and mean dose of 60 Gy. In contrast, the Peacock data and model analyzed in the previous section show that the DVHs are not symmetric in dose or volume about the mean or prescription dose (the prescription dose for Peacock plans is usually defined between 85 and 90% of the maximum target dose). With respect to the mean target dose, the underdosed tumor volume is typically smaller than the volume overdosed. Furthermore, the relative difference between the minimum and mean dose is larger than between the maximum and mean dose. Less tumor volume is underdosed but to a greater extent in dose relative to the volume overdosed. The minimum dose decreases very rapidly with standard deviation and approaches zero near SD=6 Gy, whereas, the sigmoidal distributions show that the minimum dose of the target volume decreases linearly with standard deviation and is approximately 30 Gy at SD=12 Gy. In general the minimum dose to the tumor of a nonsigmoidal distribution is smaller than that of a sigmoidal CDVH for a corresponding standard deviation.

In addition to the qualitative differences seen between the two sets of CDVHs, there is a significant difference in EUD behavior between each data set. It was reported by Niemerko that the EUD of the sigmoidal distribution decreases linearly with standard deviation and is insensitive to α and β. However, results presented in Fig. 3 show that EUD of the modeled CDVHs varies nonlinearly with respect to the standard deviation and is very sensitive to the linear‐quadratic model parameters. Depending on the degree of inhomogeneity, a 25% change in a can result in a corresponding EUD variation of approximately 40%. Consequently, EUD is unable to concisely summarize these 3D dose distributions as a single parameter.

**Figure 6:**
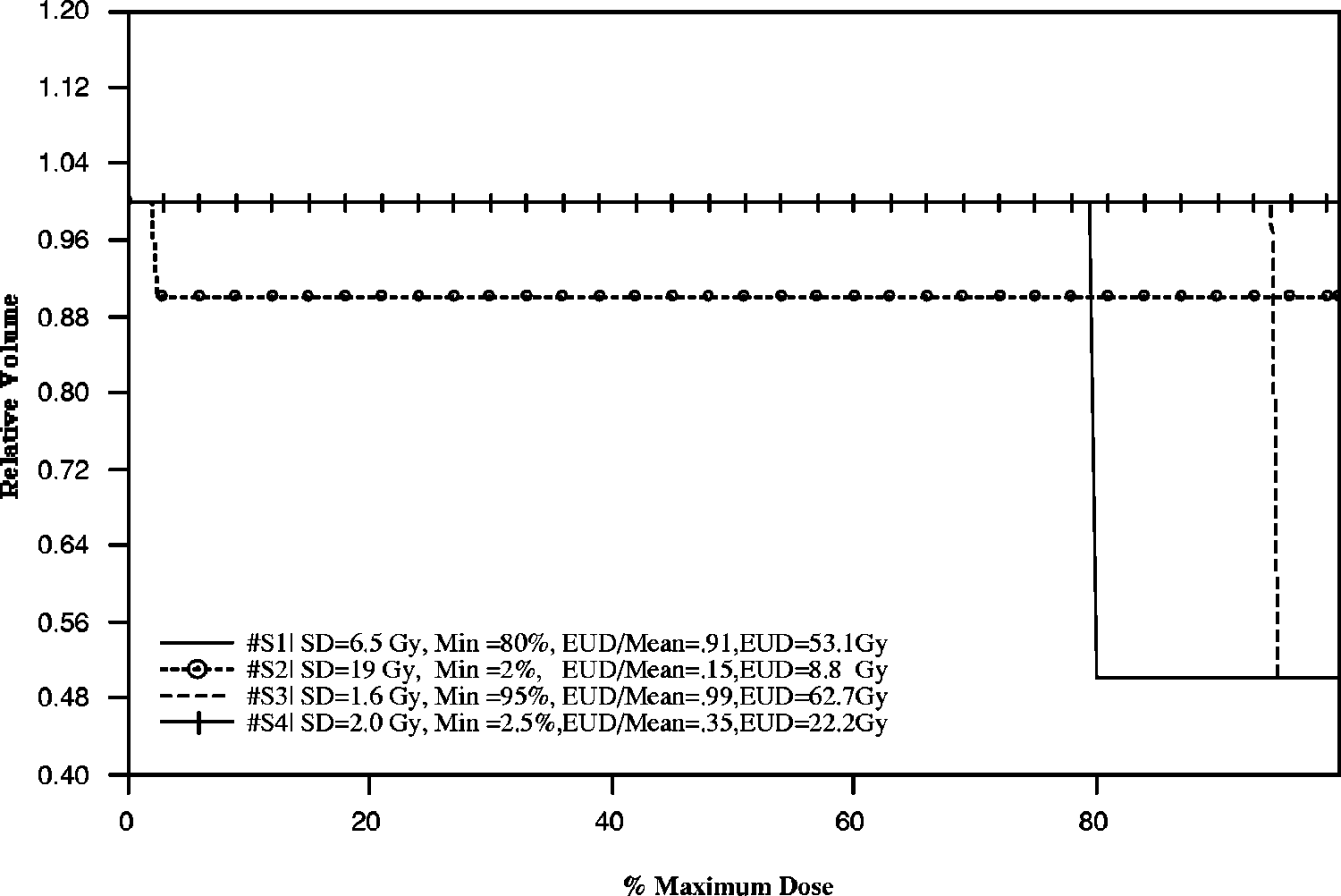
Step function examples demonstrating the differences in EUD due to dose‐volume effects. Even though standard deviation is small, EUD may be very small relative to the mean dose. In addition, EUD can have very different values for the same standard deviation. The volume effects on EUD are seen in #*S*2 and #*S*4 where the different volumes are associated with the minimum dose. EUD is calculated with α/β=10 and α=0.3.

To help understand some of the differences between dose distributions, a set of simple examples are examined in terms of equivalent uniform dose and dose statistics. Figure 6 is a graph of 4 CDVHs, each modeled using two step functions with varying minimum dose and corresponding volume. The maximum dose is the same for each distribution, 65 Gy, and the minimum dose is greater than zero to eliminate an untreated target volume element for analysis. The difference between these CDVHs is determined by the minimum dose and corresponding volume fraction — this also defines the volume fraction receiving the maximum dose. For each CDVH, EUD is calculated using 30 fractions with α=0.3 and α/β=10. By visual inspection, CDVH #*S*3 and #*S*4 appear to be approximately the same, both curves appear to be a single step function with a minimum dose near the maximum dose. Contrary to its appearance, #*S*4 has a minimum dose of 2.5% of the maximum dose, confirming that qualitative examinations of cumulative dose distributions are not sufficient for comparisons. The dose gradient for #*S*4 is large yet the standard deviation is small, 2 Gy, which suggests that standard deviation is not a good single indicator of dose inhomogeneity for distributions in general. The dose inhomogeneity is large and the corresponding EUD, ~22 Gy, is much smaller than the mean dose. CDVH #*S*4 has a large volume fraction that receives the maximum dose, 99.9%, and a small amount of relative volume receiving a minimum dose of 1.6 Gy. In comparison, #*S*3 is a dose distribution where 50% of the volume receives a large minimum dose, 63 Gy, and the equivalent uniform dose is approximately equal to mean dose due to the uniformity within the target volume. The corresponding standard deviation and dose gradient are small. These two examples show that there are conditions where that the equivalent uniform dose may be very different between distributions represented by the same standard deviation and mean dose. In addition, the standard deviation may not be an accurate predictor for inhomogeneity, and that the equivalent uniform dose should not be assumed, in general, to be the mean dose based upon the standard deviation.

According to Eq. (4), the total survival fraction for an inhomogeneous dose distribution depends on the sum of relative volume weighted survival fractions. In the step function examples, the total survival fraction depends on the contributions from the volume elements that receive the minimum and maximum dose. As an example, #*S*4, the corresponding survival fraction for a uniform minimum dose of 1.6 Gy, SF (1.6 Gy), is 0.6 and the survival fraction for the maximum dose, SF(65 Gy), is $5\times 10^{-11}$. Since the fraction of total volume receiving the minimum dose is on the order of 0.1%, the EUD is determined primarily by the minimum dose to the target. The total survival fraction of the dose distribution is $\sim 6\times 10^{-4}$ which corresponds to an equivalent uniform dose of ~22 Gy. This accounts for the fraction of total volume that receives an insufficient dose to kill a comparable fraction of cells resulting from the maximum dose. Increasing the minimum dose to the target volume will increase the number of killed cells, decrease the total survival fraction, and thereby increase the EUD. Similarly, decreasing the volume fraction irradiated to the minimum dose of 1.6 Gy, on the order of $10^{-10}$, will also decrease the survival fraction number within the volume and increase the equivalent uniform dose. This effect is also seen by comparing CDVH #*S*4 and #*S*2. The minimum dose is roughly equal between the two distributions but the fraction of volume irradiated to the minimum dose is different. In CDVH #2, 90% of the volume receives the maximum dose and 10% receives a minimum dose of 1.3 Gy. As seen in the previous comparison, the survival fraction from a low minimum dose is fairly large, SF(1.3 Gy)=0.7, and mainly determines the EUD. The equivalent uniform dose for this case is equal to 8.8 Gy which is lower than that of #*S*4 due to the larger volume fraction irradiated to an equal minimum dose. In this case, the equivalent uniform dose is larger than the minimum dose by an amount determined by the volume associated with the minimum dose.

The variation in EUD with respect to a can be understood by analyzing CDVH #*S*4. The survival fraction contributions associated with the minimum dose, 1.6 Gy, for α=0.1 and 0.5 are approximately $8\times 10^{-4}$ and $4\times 10^{-4}$, respectively. For the volume fraction, 99.9%, associated with the maximum dose of 65 Gy, the corresponding survival fractions are $\sim 3\times 10^{-4}$ and $\sim 10^{-18}$. The total survival fraction of the distribution for α=0.5 is primarily controlled by the volume receiving the minimum dose, whereas for α=0.1, the total survival fraction is determined by contributions from both volume elements. The equivalent uniform dose for α=0.5 is 17 Gy, and at the other extreme, α = 0.1, the EUD for the dose distribution is 56 Gy. The difference in EUD, with respect to α, is dependent on both, the minimum dose and the corresponding volume fraction. In situations where these factors contribute little to the overall survival fraction, EUD is insensitive to the LQ parameters. Typically this occurs for fairly uniform dose distributions where the difference between the minimum and maximum dose is small. EUD is a value between the minimum and mean dose, independent of the biological parameters, and variations decrease as the dose homogeneity increases. This is illustrated by two relatively homogeneous distributions, CDVH #*S*1 and #*S*3, where the minimum dose is 80% and 95% of the maximum dose, with the same volume fraction of 50%. In #*S*1 and #*S*3, the variation in EUD is roughly 5% and 1%, respectively.

**Figure 7:**
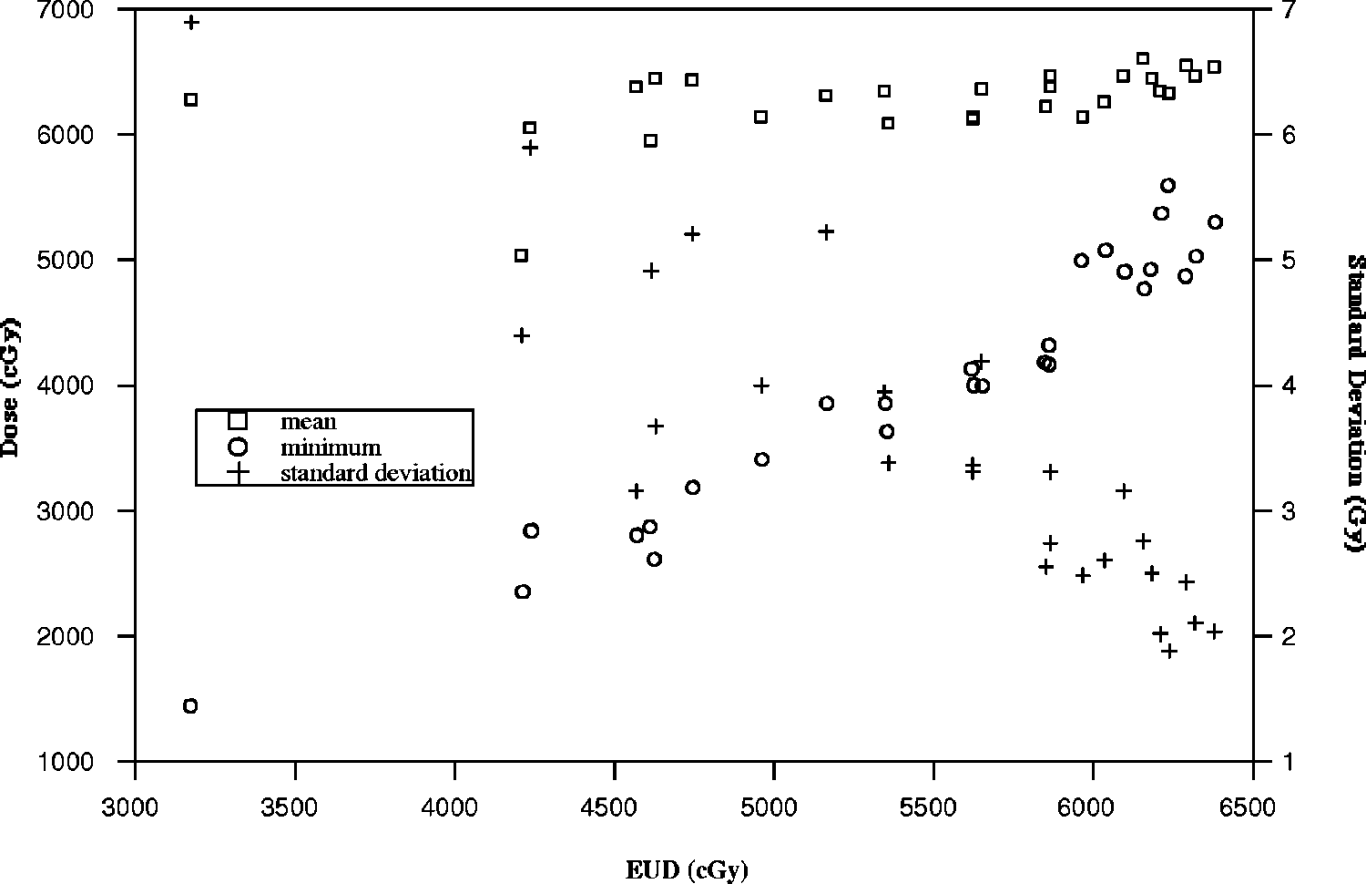
EUD for sample of Peacock patient plans, calculated for α=0.3 and α/β=10, plotted with standard deviation, mean dose, and minimum dose. EUD correlates better with minimum dose than the other statistical parameters.

From these examples, we find that the minimum and equivalent uniform dose always indicate a degree of underdosage whereas the standard deviation and mean dose do not. There are dose distributions where the mean dose is large and the corresponding EUD and minimum dose are much smaller. In other circumstances, the standard deviation is small, less than 3 Gy, and the minimum dose and EUD are much less than the mean dose. A similar correlation between EUD and the commonly quoted statistical parameters seen in the previous examples is also demonstrated by clinical data. Figure 7 shows a sample of 27 Peacock patient plans described in terms of EUD, minimum and mean dose, and standard deviation. For about half the cases, the mean dose is much larger than the corresponding EUD and minimum dose. Within the data set, the standard deviation correlates well with EUD for standard deviation values less than ~2 Gy where EUD variations are less than 10%. For standard deviations between 2 and 3 Gy, EUD is not well correlated as EUD varies by ~20%. In contrast, the minimum dose correlates well with the equivalent uniform dose and is approximately linear over the range extending to within ~90% of the mean dose. EUD indicates that dose distributions are not underdosed to the extent that the minimum dose implies and establishes an estimate of underdosage that relates to previous planning experience.

Within these plans, the minimum dose is useful for indicating the presence of underdosage but does not estimate biological effect. For example, a plan may have a minimum dose that is 15% below goal indicating that the tumor is underdosed, however, the value lacks any correlation to previous outcome studies or biological models to help with the analysis. With regard to reporting, the minimum dose is defined to an arbitrary volume for many treatment planning systems. The minimum dose is determined from the smallest dose calculated within a voxel, which is a relatively small volume and depends on the grid resolution. This will be different between planning systems and can be different between interpatient plans using the same planning system.

To help judge a treatment plan, the equivalent uniform dose provides a biological estimate of the tumor underdose which is an attempt to relate to previous experience. As demonstrated, EUD depends on the model parameters and the variation requires consideration for analysis. Without considering EUD variations, plans may be judged incorrectly; either underdosing may go undetected or the effect may be exaggerated. If EUD is independent of the linear‐quadratic model parameters as proposed by Niemerko, users are free to choose a value for their EUD calculations. Depending on the choice of α, the biological estimate will be considered to be approximately the mean dose value, and in other cases, the estimate will be significantly smaller. As an example, if α is chosen to be $0.2\;{\text{Gy}}^{-1}$, EUD is calculated to be approximately the mean dose for standard deviations less than 4 Gy as shown in Fig. 3. This might be interpreted as an acceptable plan on the basis of the single EUD value. However, if α is chosen to be $0.4\;{\text{Gy}}^{-1}$, EUD is calculated to be about 50% of the mean dose value. For plans with small EUD values, decisions may be influenced toward a less aggressive treatment to avoid complications. In either case, the biological effect will be interpreted incorrectly using a single EUD value.

We have discussed specific problems relating to the use of EUD for large inhomogeneities to limit misinterpretations within reports and analysis. People that use EUD should be aware of the sensitivity to the biological model parameters and should consider this for their analysis. Further caution is warranted toward the general use of EUD since it is based upon unaccepted biological models. Biological modeling is a controversial subject that continues to be debated within the literature and a consensus of the validity of these models has not been resolved.^13^ ^,^ ^14^ To further complicate this issue, the biological model parameters relating to specific tumors are not accurately known.

In view of these problems associated with EUD, it may assist with the interpretation of IMRT plans that contain inhomogeneous dose distributions. Not all IMRT plans are inhomogeneous but there are conditions that present dose inhomogeneities within the tumor regardless of the optimization algorithm. These conditions typically exist for tumors that require a very different dose than a nearby critical structure. In these situations, a homogeneous tumor dose delivery may not be possible due to the dose constraints for the structure and judgement is required to decide upon the degree of structure complication and tumor control. Within the tumor, there are cold spots but their size and corresponding dose does not directly relate to clinical data to form a decision. Without clinical data for inhomogeneous doses, models may help to indicate the severity of underdosing. In these cases, EUD provides an estimate of the biological effect by relating to previous clinical experience involving homogeneous tumor doses.

For our treatment plans, we use the equivalent uniform dose to assist our planning strategy and analyze patient data. We use linear‐quadratic values that are believed to be the appropriate values for the tumor and a range of values to represent the corresponding uncertainties. For head and neck cases, we use α=0.2, 0.3, and 0.4 with α/β=10. EUD sensitivity is not important for relatively homogeneous distributions since the mean dose is approximately equal to the EUD value for all parameters. For a relatively inhomogeneous distribution, EUD estimates the effect that a homogeneous dose may yield; these estimates are considered along with the normal structure doses and decisions are based on the relative merit of tumor control and structure complications. For plans with EUD below the desired goal, the structure doses and location of the minimum tumor dose are reviewed. If the minimum dose lies within the matchline plane, the isocenter point is considered with respect to the tumor; the isocenter may be shifted vertically to improve the dose distribution. For plans where the minimum dose is along the tumor periphery near a critical structure, the structure dose distribution is examined using CDVHs, location, and statistics. The decision to increase or decrease the tumor dose is determined by the amount of underdosing estimated from EUD and possible structure complication.

The concept of EUD relates homogeneous doses to nonuniform dose distributions through survival fraction calculations. We have restricted the discussion to tumor types that were routinely treated with uniform doses according to ICRU 50. The emphasis has been directed toward relating inhomogeneous doses resulting from IMRT, where clinical data is unavailable, to homogeneous doses where clinical experience and data are available. There may be other treatment procedures for consideration such as brachytherapy or stereotactic radiosurgery. In either of these specialties, however, the clinical experience is with inhomogeneous doses and EUD might be regarded as a scoring parameter between inhomogeneous plans. Where the minimum dose to the tumor is specified, EUD indicates the degree of hot spots that may be useful for comparing plans.

## CONCLUSION

We have discussed specific problems related to EUD for large inhomogeneities to prevent misinterpretations when using the concept for reporting and analysis. People using EUD should be aware of the sensitivity to the biological model parameters and should consider this during treatment plan evaluations. Previous recommendations state that a single value of EUD, independent of the model parameters, is sufficient for reporting and analysis. Contrary to this, the equivalent uniform dose, based on the linear‐quadratic model, is not in general independent of the model parameters, α and β. Using a single EUD value may lead to compromising tumor control or increased normal tissue complications; a small EUD value may bias the judgment toward a more palliative treatment or a large value may lead to underdosing. Proper analysis should include the appropriate model parameters for the tumor and a range of uncertainties associated with those values.

In comparison with the statistical parameters used for describing dose distributions for reporting, EUD may exhibit some advantages. The minimum dose has been recommended for reporting large dose inhomogeneities without specifying a corresponding volume. Conversely, EUD includes both volume and dose. EUD may be a more accurate parameter for outcome correlations due to the sensitive dose‐volume relation defined by the biological model equation. Again, the variation in EUD with respect to the model parameters needs consideration before reporting dose distributions.
